# Supplementary material for: The Anti-Inflammatory Effect of Nanoarchaeosomes on Human Endothelial Cells
Source: Pharmaceutics. 2022 Mar 29;14(4):736. doi: 10.3390/pharmaceutics14040736 (PMC9027062; doi:10.3390/pharmaceutics14040736)
Supplement: Supplementary file 1 [file pharmaceutics-14-00736-s001.zip › pharmaceutics-1630045-supplementary.pdf]

pharmaceutics

Article

The Anti-Inflammatory Effect of Nanoarchaeosomes on Human Endothelial Cells

Nancy Charó, Horacio Jerez, Silvio Tatti, Eder Lilia Romero and Mirta Schattner

Table S1. List of Primers sequences.

Eef1A1 Fwd	TCGGGCAAGTCCACCACTAC
Eef1A1 Rv	CCAAGACCCAGGCATACTTGA
ICAM-1 Fwd	GACTCCAATGTGCCAGGCTT
ICAM-1 Rv	TAGGTGCCCTCAAGATCTCG
vWF Fwd	GTTGTGGGAGATGTTTGCCTAC
vWF Rv	TTTACCTCCCTCAGCCAGACA

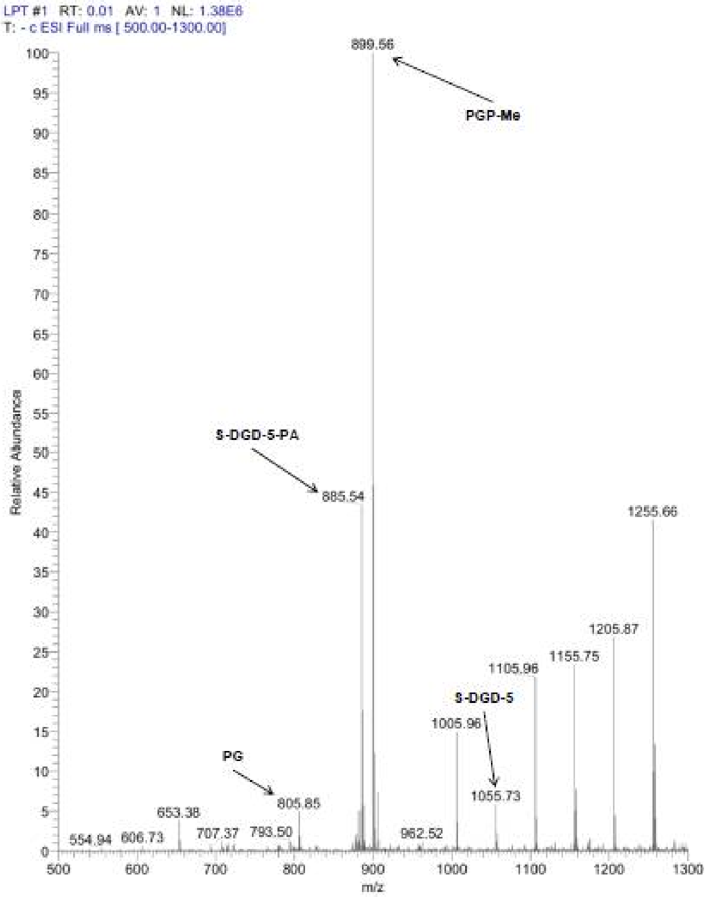

Figure S1. ESI- MS of TPL from *H. tebenquichense.* TPA were dissolved to 1 mg/ml in chloroform:methanol 1:1 v:v and filtered through 0.22 µm nylon membranes. Spectra were recorded using a Thermo Finnigan LCQ Ion Max equipped with an electrospray ionization source. Samples were continuously fed into the spectrometer at a flow rate of 10 µl/min. The interface conditions were: Nebulizer gas (air) 12 l/min, cone gas (nitrogen), needle voltage 1.2 l/min, -5.0 kV (negative ions), and mass range 50-2000 uma. TPA samples were diluted 1/1000 and analyzed using ESI-MS in negative mode to detect archaeolipid signals under the conditions described previously.

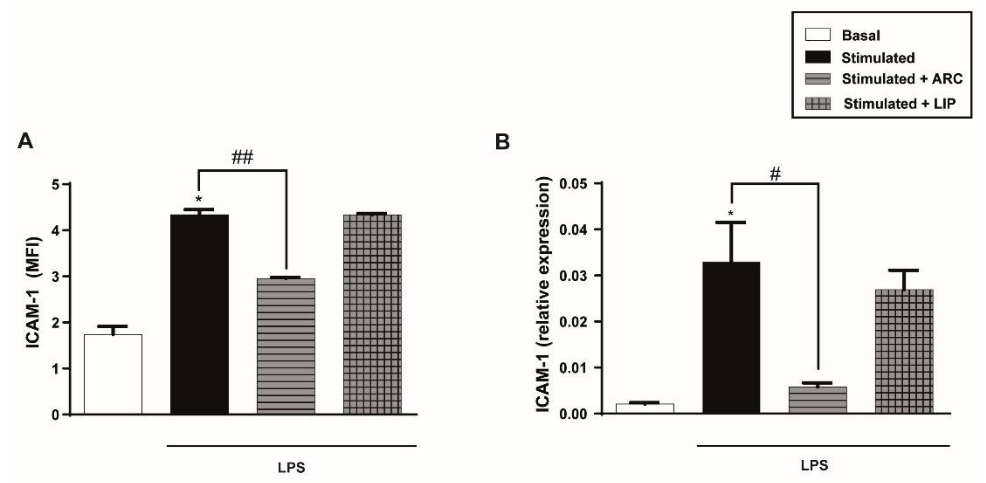

Figure S2. ARC selectively inhibit the protein and mRNA expression level of ICAM-1 on stimulated endothelial cells. HMEC-1 were stimulated with LPS (1 µg/ml) in the absence or presence of nanovesicles for 18h. A) ICAM-1 expression was evaluated by flow cytometry 18h poststimulation and B) mRNA expression levels of ICAM-1 were evaluated by qPCR and referred to as 2^ delta cycle threshold (dCT). Eef1A was used as the housekeeping gene (One-way ANOVA followed by the Bonferroni multiple comparisons test. *P < 0.05 vs Basal; #P < 0.05 and ##P < 0.01 between the marked groups. The results are the mean ± SEM of 4 to 5 independent experiments.

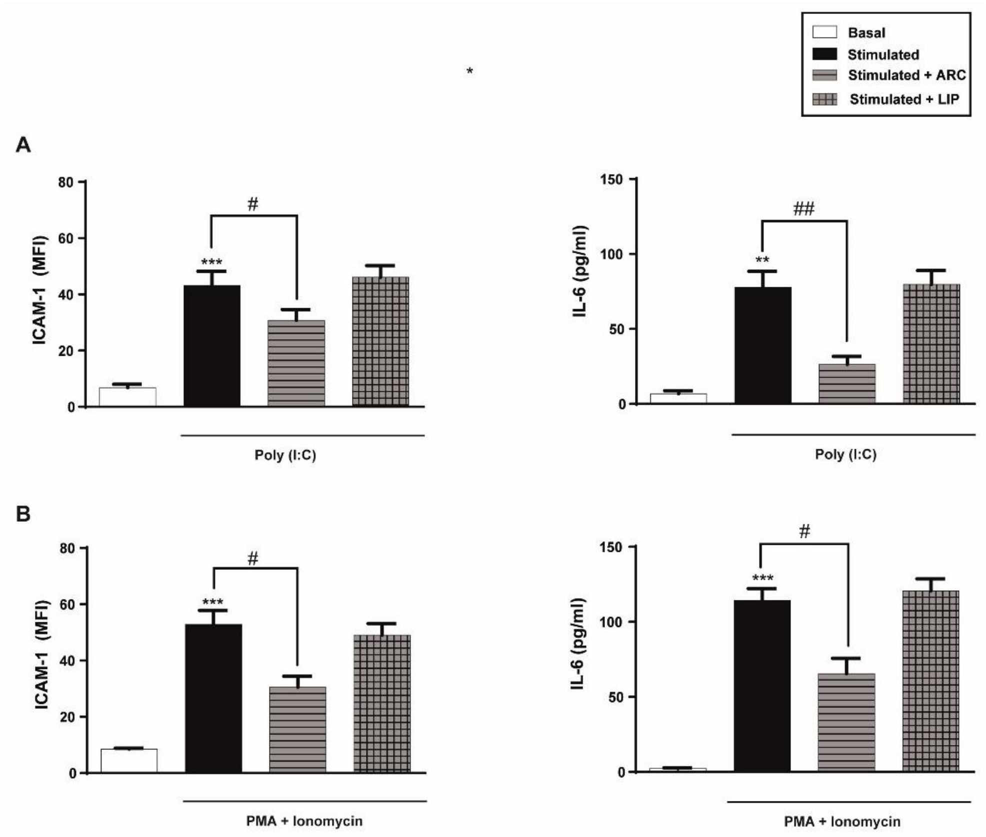

Figure S3. ARC inhibit the expression of ICAM-1 and secretion of IL-6 on stimulated endothelial cells. HUVEC were stimulated with (A) Poly (I:C) (1 µg/ml) and (B) PMA (10 nM) + Ionomycin (1 µg/ml) and incubated with archaeosomes (ARC) or liposomes (LIP) at 50 µg/ml for 18h. ICAM-1 expression was evaluated by flow cytometry 18 h poststimulation and levels of IL-6 in the supernatants were determined by ELISA (One-way ANOVA followed by the Bonferroni multiple comparisons test; **P < 0.01 and ***P < 0.001 vs Basal; #P < 0.05 and ##P < 0.01 between the marked groups). The results are the mean ± SEM of 4 to 5 independent experiments.

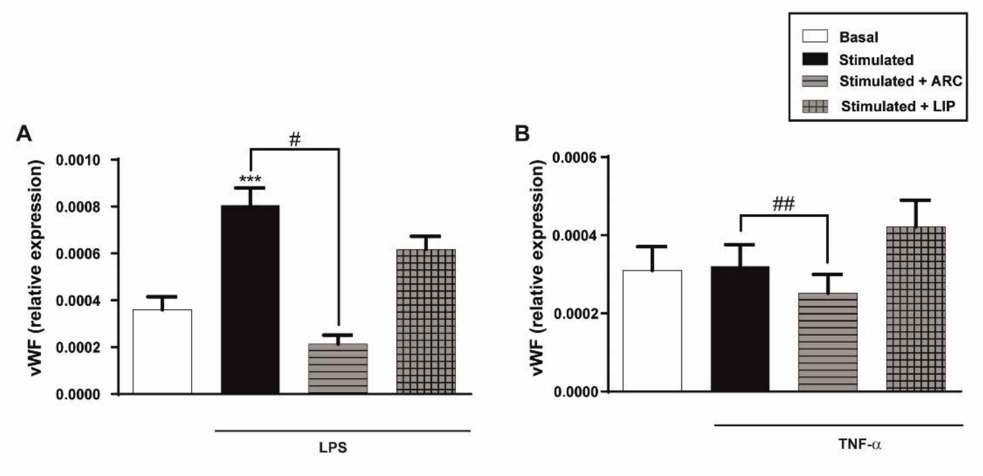

Figure S4. ARC inhibit the mRNA expression level of vWF on stimulated endothelial cells. HMEC-1 were stimulated with (A) LPS (1 μg/ml) or (B) TNF-α (3 ng/ml) in the absence or presence of nanovesicles for 18h (One-way ANOVA followed by the Bonferroni multiple comparisons test; ***P < 0.001 vs Basal; #P < 0.05 and ##P < 0.01 between the marked groups). The results are the mean ± SEM of 4 to 5 independent experiments.

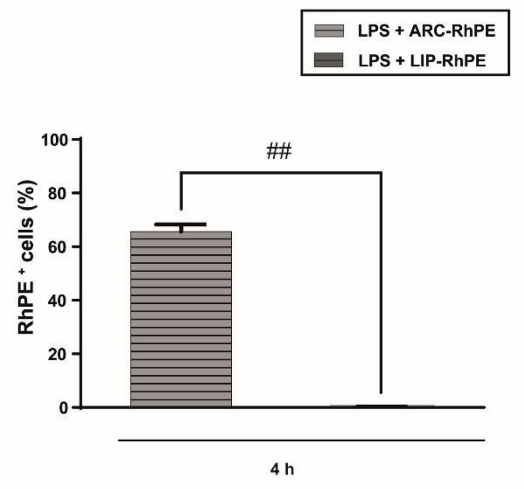

Figure S5. ARC are internalized by stimulated HUVEC. HUVEC were stimulated with LPS (1 μg/ml) in the absence or presence of RhPE-labeled nanovesicles (ARC-RhPE and LIP-RhPE) for 4h. Uptake studies were performed by flow cytometry (Two-sided Student's paired t test; ##P < 0.01 between the marked groups). The results are the mean ± SEM of 4 to 5 independent experiments.
